# The shield of the screen: The role of anger on the development of social media addiction and internet gaming disorder

**DOI:** 10.3934/publichealth.2024051

**Published:** 2024-09-24

**Authors:** Fiammetta Iannuzzo, Clara Lombardo, Maria Catena Silvestri, Fabrizio Turiaco, Giovanni Genovese, Caterina Rombolà, Carmela Mento, Maria Rosaria Anna Muscatello, Antonio Bruno

**Affiliations:** 1 Department of Biomedical and Dental Sciences and Morphofunctional Imaging, University of Messina, Messina, Italy; 2 Department “Scienze della Salute”, University of Catanzaro, Catanzaro, Italy; 3 Psychiatry Unit, Polyclinic Hospital University of Messina, Messina, Italy

**Keywords:** internet addiction, social media addiction, internet gaming disorder, anger

## Abstract

This study aims to explore if the dimensions of anger can be risk factors for the development of social media addiction and an internet gaming disorder, considering that the correlation between anger and these specific subcategories of internet addiction can represent a core intervention in their prevention and treatment. 477 subjects, recruited among the general population, were assessed on-line by the following tools: STAXI–2; BSMAS, and IGDS9–SF. A correlation analyses showed a significantly positive relationship between the total score of the BSMAS and the STAXI–2 scales SANG (*p* < 0.0001), TANG (*p* < 0.0001), AX–O (*p* = 0.003), and AX–I (*p* < 0.0001), and between the total score of the IGDS9–SF and the STAXI–2 scales SANG (*p* = 0.002), TANG (*p* < 0.0001), AX–O (*p* = 0.001), AX–I (*p* < 0.0001), and AC–O (*p* = 0.004). A linear regression model showed how TANG and AX–I were direct predictors of the BSMAS total scores, and how AX–I was a direct predictor of the IGDS9–SF total scores. It appears plausible that there is a significant correlation between the dimensions of anger and the emergence of social media addiction and internet gaming: internet usage may serve as a coping mechanism for emotional or social challenges and as a protective screen to deal with negative emotions.

## 1. Introduction

In the digital era, technology contaminates every sphere of life: virtual connections are increasingly becoming a tool of social interaction, in which play spaces are shifting to constantly evolving online platforms and many daily activities are shaped and implemented through digital hybridization. In 2023, the number of internet users worldwide stood at 5.3 billion, which equates to around two thirds of the global population being currently connected to the world wide web [1]. Alongside the many positive aspects that digital life has brought with it, there are also negative aspects; for a minority of people, the internet can lead to the development of an addiction characterized by tolerance, backwardness, emotional disturbances, and disrupted social relationships [2]. Currently, there is no officially recognized definition of internet addiction; however, this category appears to include several subcategories of disorders (e.g., addiction to social media, online gaming virtual sex or virtual relationships, online gambling, compulsive online shopping, and cognitive overload) [3],[4].

Previous studies have revealed the negative impacts of social media abuse on a users' health; although using social media in a detrimental manner is not yet formally categorized as an addiction, most studies have explored the relationship between social media use and its addictive effects, which raised concerns surrounding social media addiction (SMA) [5]. Though criteria for the diagnosis of SMA have not formally been established, existing validation methods using questionnaires are based on the interpolation of classic symptoms for behavioral and substance addictions derived from the component model of addiction created by Griffiths [6].

Within this field, only the problematic use of video games, namely internet gaming disorder (IGD), is officially recognized as a mental disorder placed in the third section of the Diagnostic and Statistical Manual of Mental Disorders, fifth version (DSM5) [7], and is a condition that requires further research and clinical investigations. According to DSM, the diagnosis of IGD needs five of the nine diagnostic criteria (preoccupation or obsession, withdrawal, tolerance, loss of control, loss of interest, excessive and continued use, deception, escape from negative feelings, and functional impairment) to be reported over the course of one year.

Prospective research has analyzed predisposing factors to internet addiction; inadequate stress-coping strategies, psychological distress, depression, and other psychopathological issues seem to be involved into the genesis of this kind of new addictions [8],[9], and is most frequently associated with several other psychopathological issues, such as depression self-destructive behaviors, anxiety, attention deficit/hyperactivity, hostility/aggression, obsessive-compulsive symptoms, relational problems, and eating disorders [10]. However, no study seems to have explored the specific role of anger in the development of internet addiction, even though impulsivity and aggression have been explored in some research attempts [11].

In the neurobiological and behavioral sciences, anger is an emotion that consists of feelings that vary in intensity, from mild irritation or annoyance to intense fury and rage [12], encompasses various brain regions and specific hormonal signals, and is influenced by genetic and environmental factors that affect its manifestation [13],[14]. The way anger is expressed can greatly differ among individuals; some may express their anger outwardly through screaming, physical aggression, or other evident signs, while others may internalize their anger, leading to avoidance or passive-aggressive behaviors. The mode of expression can influence both personal well-being and interpersonal relationships and can also reflect different experiences across cultural contexts. From a psychological point of view, anger was analyzed in many of its components: state anger and trait anger are the main dimension, which can also be declined into specific subcomponents. State anger refers to an emotional state characterized by active feelings of varying intensity [15],[16], while trait anger is the tendency to react to frustrating situations with a frequent increase in anger [15],[16].

According to some studies, anger is generally associated with a lower quality of life, a broad and heterogeneous spectrum of diseases, and high-risk addiction behaviors, such as smoking habits, alcohol, and drug abuse, that are high social impact conditions [17]–[19].

The relationship between aggressive behaviors and internet addiction has been explored in previous studies [20], as well as the connection between SMA and IGD and specific existential conditions such as loneliness related to substance use disorders [21]; however, no study has investigated the specific association amongst SMA, IGD, and anger.

Based on the previous background, the aim of the present study is to explore if the dimensions of anger can be risk factors for the development of SMA and IGD, taking into account that the correlation between anger and these specific subcategories of internet addiction can represent a core intervention in their prevention and treatment.

## 2. Materials and methods

### 2.1. Sample and procedures

This study was conducted on a healthy general population (18–65 years) using the recruitment method applied in our previous research on internet addiction: the selection procedures and the inclusion and exclusion criteria can be consulted in the specific “Materials and methods” section [18].

The priority sample size estimation was calculated by assuming a population size of 51.5 million, a margin of error of 5%, a confidence level of 95%, and a standard deviation of 50%; from these criteria, a sample size of 386 was determined.

Data were collected between January and March 2022. Since the study was observational (non-invasive and non-interactive), completely anonymous, and was conducted on healthy voluntary subjects (i.e., “not vulnerable”), ethical approval was not mandatory.

### 2.2. Measures

The following instruments were used to assess anger and the problematic use of online video games and social media:

- State - Trait Anger Expression Inventory-2, STAXI–2 [15]- Italian version: a 57-item self-report inventory which uses 4-point Likert scales to measure the intensity of anger as an emotional state (state anger; SANG), how the individual is disposed to angry feelings as a personality trait (trait anger; TANG), anger expression outward (AX–O) and inward (AX–I), anger control outward (AC–O) and inward (AC–I), and the anger expression index (AX). Raw scores on STAXI–2 scales are converted to sex- and age-specific T-scores and percentile scores established in the initial validation of the scale. T-scores above 60 and below 40 are considered to fall outside the normative range. The internal consistency reliability has a value of α ranging from 0.73 to 0.95 for the total scale and from 0.73 to 0.93 for the subscales.

- Bergen Social Media Addiction Scale (BSMAS): the Italian version of the BSMAS scale assesses social media use over the past year. It consists of six items that reflect the basic elements of addiction (salience, mood modification, tolerance, withdrawal, conflict and relapse) according to Griffith's 89 model. Each item deals with experiences over a 12-month time span to be answered on a 5-point Likert scale ranging from 1 (very rarely) to 5 (very often). The final scores range from 6 to 30, where higher scores indicate a greater risk of SMA. A cut-off points of 24 indicates a pathological score. The internal consistency for the Italian version was examined using Cronbach's α, which was very positive (*α* = 0.88) [22].

- Internet Gaming Disorder Scale-Short-Form (IGDS9–SF): the Italian version of the IGDS9–SF assesses the severity of online gaming addiction (IGD) and its effects by examining both online and offline gaming activities performed over a 12-month period. The scale includes nine items that correspond to the nine diagnostic criteria of IGD as defined by the American Psychiatric Association's DSM–5 [7]. The responses are broken down on a 5-point Likert scale ranging from 1 (never) to 5 (very often), with scores ranging from 9 to 45, where higher scores indicate a higher degree of IGD. A cut-off points of 21 indicates a pathological score [23]. The IGDS9-SF had an excellent reliability with an internal consistency coefficient (Cronbach's *α*) of 0.96 [23].

### 2.3. Statistical analysis

Continuous and non-continuous data were expressed as (*mean* ± *standard deviation*) or as a percentage, when appropriate. A partial correlational analysis controlled for age was carried out to assess the possible relationships between anger, social media, and gaming addiction. Finally, the BSMAS and IGDS9–SF total scores (dependent variables) and the STAXI2 scales (independent variables) were entered into two linear regression models to explore a possible anger dimensions role as predictors of social media and gaming addiction. To lessen the risk of Type 1 errors, a Bonferroni correction for multiple comparisons was achieved (0.05/8) and a value for *p* ≤ 0.006 was selected as significant.

## 3. Results

511 complete surveys were received and 76 were excluded according to the selected inclusion/exclusion criteria. The final sample included 435 subjects (33.8% male), with an average age of (32.9 ± 11.6) years, and a high school qualification or higher in 97.4% of the cases. The demographic and clinical features of the total sample are shown in Table 1. Regarding the STAXI–2 questionnaire, the results showed in the sample examined mean scales scores within the normal range. Concerning the BSMAS scale, the results highlighted moderate values of the problematic use of social media in the sample examined; 22 subjects (4.8%) of the total sample reported clinically significant scores. Finally, regarding the IGDS9–SF questionnaire, total scores indicative of a mild level of the problematic use of video games emerged in the sample examined; 32 subjects (7.4%) of the total sample reported clinically significant scores.

Table 2 reports the partial correlation analyses (controlled for age) carried out to evaluate the possible associations between anger, social media, and gaming addiction: the data highlighted a direct association between the BSMAS “total score” and the STAXI–2 scales “SANG” (*p* < 0.0001), “TANG2 (*p* < 0.0001), “AX–O” (*p* = 0.003), and “AX–I” (*p* < 0.0001), and between the IGDS9–SF “total score” and the STAXI–2 scales “SANG” (*p* = 0.002), “TANG” (*p* < 0.0001), “AX–O” (*p* = 0.001), “AX–I” (*p* < 0.0001), and “AC–O” (*p* = 0.004).

Subsequently, the STAXI2 scales (as independent variables) were entered in two linear regression models to assess the potential role as explanatories towards the BSMAS and IGDS9–SF total scores (as dependent variables) (Tables 3): as a block, the statistical models accounted for 12.1% and 8.7% of the total variance, respectively, in the “BSMAS total scores” (*F* = 9.787; *df* = 6; *p* < 0.0001) and the “IGDS9–SF total scores” (*F* = 6.839; *df* = 6; *p* < 0.0001). Data obtained showed that only “TANG” (*β* = 0.195; *p* = 0.003) and “AX–I” (*β* = 0.259; *p* < 0.0001) were direct predictors of the “BSMAS total scores”, and “AX–I” (*β* = 0.202; *p* < 0.0001) was a direct predictor of the “IGDS9–SF total scores”; conversely, the other STAXI2 scales were not significant in predicting social media and gaming addiction.

**Table 1.** Demographic and clinical features of the final sample (*n* = 435).

Project	Total group
Gender (M/F) (%)	33.8/66.2
Age (*Mean* ± *S.D*.)	32.97 ± 11.57
Educational level (%)	
Elementary School	0.2
Middle School	2.3
High School	43.4
Degree	50.3
Postgraduate degree	3.7
STAXI2 (*Mean* ± *S.D*.)	
SANG	52.78 ± 13.33
TANG	50.61 ± 11.72
AX–O	51.26 ± 11.93
AX–I	55.80 ± 12.10
AC–O	51.87 ± 10.40
AC–I	52.71 ± 10.60
BSMAS Total score (*Mean* ± *S.D*.)	14.80 ± 5.15
IGDS9–SF Total score (*Mean* ± *S.D*.)	11.78 ± 4.95

**Table 2.** Partial Correlation analysis (controlled for age).

Project	SANG	TANG	AX–O	AX–I	AC–O	AC–I
BSMAS Total score	0.171**	0.234**	0.140*	0.294**	-0.027	-0.023
IGDS9–SF Total score	0.144*	0.201**	0.165*	0.220**	-0.138*	-0.096

Note: * *p* < 0.006; ** *p* < 0.0001.

**Table 3.** Linear regression analysis.

**Project**	**Predictors**	**Unstandardized coefficients**		**Standardized coefficients**	***t***	***p***
**Dependent Variable**		B	S.E.	Beta		
**BSMAS Total score ^a^ (Model 1)**	(Constant)	4.410	2.208	-	1.997	0.046
	SANG	0.012	0.020	0.032	0.596	0.552
	TANG	0.086	0.028	0.195	3.034	0.003
	AX–O	-0.020	0.025	-0.047	-0.802	0.423
	AX–I	0.110	0.021	0.259	5.265	0.000
	AC–O	-0.005	0.033	-0.011	-0.161	0.872
	AC–I	0.011	0.030	0.022	0.348	0.728
**IGDS9–SF Total score ^b^ (Model 2)**	(Constant)	6.901	2.164	-	3.188	0.002
	SANG	0.013	0.020	0.034	0.632	0.527
	TANG	0.039	0.028	0.091	1.394	0.164
	AX–O	0.009	0.025	0.021	0.358	0.720
	AX–I	0.083	0.021	0.202	4.018	0.000
	AC–O	-0.060	0.032	-0.125	-1.867	0.063
	AC–I	0.006	0.030	0.012	0.186	0.853

Note: ^a^ *R* = 0.347; *F* = 9.787; *p* ≤ 0.0001; ^b^ *R* = 0.296; *F* = 6.839; *p* ≤ 0.0001.

## 4. Discussion

This study was designed to evaluate if the dimensions of anger could be risk factors for the development of SMA and IGD.

The prevalence rate of problematic social media use in the examined sample was 4.8%, which is consistent with literature that indicated a 5% estimated prevalence for SMA in the general population [24]; conversely, problematic video game use in our sample was 7.4%, which surpasses the estimated 4.7% prevalence of IGDs among the general population [25].

From the correlation analyses, we observed a statistically significant direct association between the BSMAS total score, the STAXI–2 scales SANG, TANG, AX–O, and AX–I, and between the IGDS9–SF total score and the STAXI–2 scales SANG, TANG, AX–O, AX–I, and AC. Based on our data, the two types of addiction are directly associated with various dimensions of anger, suggesting a potential role of anger in the onset of these types of addiction. This supports existing literature which indicates a possible connection between anger and addictive behaviors [26].

Linear regression models underline how trait anger and AX–I were direct predictors of the BSMAS total scores, moderately the first and strongly the last, and how AX–I was a strong direct predictor of the IGDS9–SF total scores. First, the results of the linear regression showed how trait anger seems to induce a problematic use of social media, while no correlations emerged between state anger and SMA. This outcome disagrees with literature which suggests state anger as a factor involved in the genesis of addiction because of the observed tendency of people to turn the social media as an escaping strategy to alleviate dysphoric and negative moods and to satisfy psychological needs [26]–[28]. Although no strong correlations have been observed between trait anger and addictions in literature, anger is a negative emotion, and there is evidence that confirms the relationship between negative emotions and addiction development [29].

Second, the result of the regression model shows the factor of AX–I as strongly predictive of both IGD and SMA. The AX–I subscale of STAXI–2 measures the extent to which an individual ‘holds things in’ or suppresses anger and includes items such as “when I feel angry, I boil inside but don't show it” [28]. The development of IGD and SMA, caused by the tendency to direct anger inward, could be explained as the result of maladaptive strategies in individuals with angry traits. Maladaptive coping strategies which involve anger have already been observed in other pathological patterns such as depression [30]. According to psychoanalytic theorists, inwardly directed anger leads to feelings of guilt and contributes to the development of depression, and research has shown that depressed individuals tend to suppress their anger and are more likely to involve self-blame or the act of blaming others [31],[32]. Consistent with literature, self-blame, blaming others, rumination, and catastrophizing can predict the symptoms of internet addiction [33].

**Figure 1.**
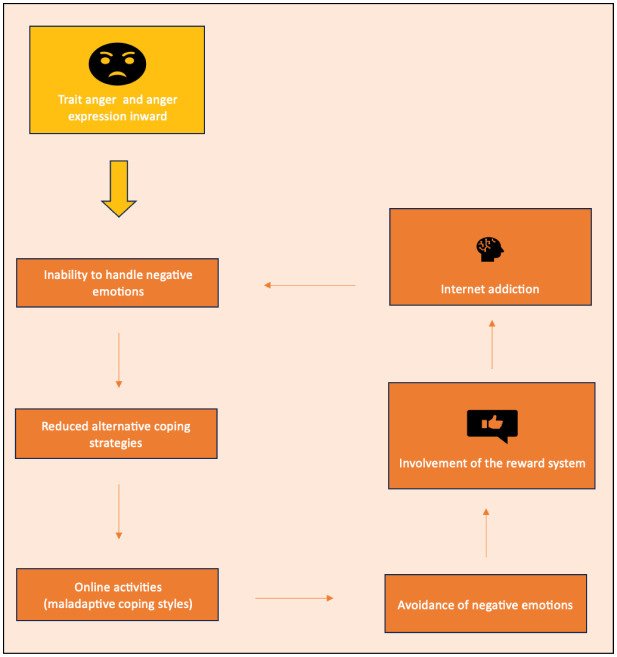
The circle of addiction triggered by dimensions of anger.

Considering our results, it appears plausible that there is a significant correlation between the dimensions of anger and the emergence of SMA and IGD: internet usage may serve as a coping mechanism for emotional or social challenges, and as a protective screen to deal with negative emotions. When online activities are excessively utilized to deal with negative emotions, and when alternative coping strategies diminish, the reliance on online activities to evade negative feelings may eventually culminate in an internet addiction (Figure 1).

In this context, individuals with a maladaptive coping style, and those who expect to use the internet to modify their mood, may have a higher likelihood of developing a dependence on it [34].

The current study has some limitations to note. First, the sampling method (an online voluntary survey on internet usage) might have introduced a notable self-selection bias toward individuals with elevated levels of problematic internet use, as evidenced by the noted high prevalence of IGD in our sample. Additionally, the correlational nature of the study prevented the prediction of causality between the variables and did not rule out the potential influence of third variables. Moreover, employing self-assessment measures did not eliminate the likelihood of defensive response patterns and limited self-awareness, which potentially impacts the reliability of the responses, even considering that the chosen tests do not include control items, making it difficult to verify whether the recruited subjects responded in a systematic manner. Finally, gender differences were not considered in the expression of anger.

## 5. Conclusions

Despite the limitations, this is the first study to explore the role of anger in the development of SMA and IGD. The pervasive influence of technology in our daily lives raises the issue of addressing the emerging forms of behavioral addictions, such as internet addiction. The literature suggests the difficulty in managing and overcoming addiction, which underscores the importance of exploring novel therapeutic approaches for early intervention. Considering anger as a potential contributing factor to the emergence of some kind of internet dependencies is an opportunity for enhanced diagnoses and treatments. Nevertheless, more comprehensive research is necessary to fully understand this phenomenon, and further studies on potential gender disparities in the relationship between anger and internet addiction could offer other insights into personalized and gender-specific intervention strategies.

## Use of AI tools declaration

The authors declare they have not used Artificial Intelligence (AI) tools in the creation of this article.
